# Effectiveness and Safety of Roflumilast in the Treatment of Psoriasis: A Systematic Review and Meta-Analysis

**DOI:** 10.2174/0115748863406187251031101244

**Published:** 2026-02-10

**Authors:** Ali Mohammed Aleid, Houriah Yasir Nukaly, Salma Mohammed Alshreef, Mishari Tariq Alrubaiaan, Shahad Hamoud Alrowais, Mohammed Khalid Alghamdi, Shatha Saleh Al Ghamdi, Laila Mohammed Alali, Mohammad A. Alghamdi, Mahdi Al Dhafiri

**Affiliations:** 1 Dermatology Department, Ministry of Health, King Faisal University (KFU), Moleer Clinic, Riyadh , Saudi Arabia; 2 Medicine Program, Batterjee Medical College, Jeddah 21442 , Saudi Arabia; 3 Umm Al-Qura University, Makkah , Saudi Arabia; 4 King Saud Bin Abdulaziz University for Health Sciences, College of Medicine, Riyadh , Saudi Arabia; 5 Princess Nourah Bint Abdulrahman University, College of Medicine, Riyadh , Saudi Arabia; 6 University of Jeddah, College of Medicine, Jeddah , Saudi Arabia; 7 King Abdulaziz University, College of Medicine, Jeddah , Saudi Arabia; 8 Jazan University, College of Medicine , Jazan 82911m Saudi Arabia; 9 Internal Medicine Department, Division of Dermatology, Faculty of Medicine, Al-Baha University, Al-Baha , Saudi Arabia; 10 Division of Dermatology, College of Medicine, King Faisal University, Al-Ahsa , Saudi Arabia

**Keywords:** Erythema, phosphodiesterase inhibitors, psoriasis, roflumilast, chronic autoimmune, plaque psoriasis

## Abstract

**Background:**

Psoriasis, a chronic autoimmune condition, can severely impact patients' well-being. It is characterized by erythema, thickening, and scaling of the skin. Plaque psoriasis, the most common subtype (80%-90% of cases), varies from localized to severe forms. Although corticosteroids are commonly used to treat psoriasis, prolonged use poses risks. Therefore, alternative therapies are needed. Roflumilast, a potent phosphodiesterase 4 inhibitor, is currently being considered as a treatment for plaque psoriasis.

**Methods:**

We searched four electronic databases (Cochrane Central Register of Controlled Trials, PubMed, Scopus, and Web of Science) up to March 2024 for relevant articles evaluating the efficacy and tolerability of roflumilast in the management of psoriasis. The quality of evidence from trials was assessed using the Cochrane Risk of Bias tool (RoB1). Data were extracted using a pre-defined template in Google Sheets and analyzed using RevMan 5.4.

**Results:**

Roflumilast significantly improved IGA success (RR = 3.48, 95% CI [2.04–5.92], *P <* 0.00001 at week 4; RR = 4.02, [3.17–5.11], *P <* 0.00001 at week 8). The pooled studies demonstrated homogeneity at both weeks 4 ( *P =* 0.17, I² = 38%) and 8 ( *P =* 0.38, I² = 5%). Regarding the results of the Psoriasis Area and Severity Index, 75% favored roflumilast over placebo (RR = 2.72, 95% CI [1.18 to 6.28], *P <* 0.00001, and RR = 3.41, 95% CI [2.19 to 5.32], *P <* 0.00001, at weeks 4 and 8, respectively). Subgroup analysis by roflumilast dose (0.15% *vs* . 0.3%) resolved heterogeneity (I² = 0%).

**Conclusion:**

This meta-analysis represents the first investigation into the efficacy and safety of roflumilast for treating psoriasis. Results suggest that roflumilast is both effective and well-tolerated in managing psoriasis. Further large-scale RCTs are warranted to confirm efficacy and long-term safety.

## 1 INTRODUCTION

Psoriasis, a chronic immune-mediated disease affecting both genders equally, impacts ~125 million globally [1]. It impacts approximately 125 million individuals worldwide [1]. The incidence of new cases is estimated at approximately 80 per 100,000 person-years [2]. Its prevalence follows a roughly linear increase over the life course, from 0.12% at the age of 1 year to 1.2% at the age of 18 years [3]. The pathogenesis of psoriasis primarily involves an autoimmune response resulting from the activation of various inflammatory cells through a cytokine activation cascade. IL–23–mediated Th17 activation drives downstream events, including keratinocyte proliferation, increased expression of angiogenic mediators and endothelial adhesion molecules, and immune cell infiltration into the skin [4, 5]. Besides genetic predisposition and aging, environmental factors, such as climate, sun exposure, and ethnic background, also contribute to psoriasis susceptibility [6].

Psoriasis is characterized by three key clinical features: (1) thickening, (2) erythema, and (3) scaling. The condition presents in various subtypes, including plaque psoriasis (psoriasis vulgaris), guttate (droplet) psoriasis, eruptive psoriasis (characterized by scaly teardrop-shaped spots), inverse psoriasis (typically found in skin folds also referred to as intertriginous or flexural psoriasis), pustular psoriasis (further classified into palmoplantar pustular psoriasis or generalized pustular psoriasis), and erythrodermic psoriasis (a rare but severe complication of any psoriasis subtype) [7]. Plaque psoriasis is the most common type of psoriasis, affecting approximately 90% of patients, which is usually presented by monomorphic, sharply demarcated erythematous plaque covered with silver lamellar scales [8]. These plaques may appear in a few large areas of the body or present as erythroderma affecting the entire body. Scalp involvement is common, observed in 75%-90% of patients [9].

The treatment of psoriasis encompasses a diverse array of therapeutic approaches. Mild or localized psoriasis is typically managed with corticosteroids, vitamin D analogues, calcineurin inhibitors, and targeted phototherapy [10]. Corticosteroids, either used alone or along with vitamin D analogues or keratolytic agents, are the cornerstone of treatment. They work by exerting anti-inflammatory and antiproliferative effects, resulting in the suppression of the previously mentioned proinflammatory cytokines [11, 12]. However, prolonged use over an extensive surface area may pose risks to the pituitary or adrenal axis. Vitamin D analogues work by binding to vitamin D receptors on keratinocytes, blocking their differentiation and proliferation. A meta-analysis has demonstrated that the combination of vitamin D analogues and topical corticosteroids is the most effective treatment [13]. Moderate to severe psoriasis may necessitate systemic treatments, including biologics and oral agents (such as methotrexate or cyclosporine), in conjunction with topical treatment or phototherapy. Biologics represent major advancements in psoriasis treatment, acting by blocking the cytokine cascade molecules. These agents include TNF-α, IL-17, and IL-12/23 inhibitors [14].

Cyclic AMP (cAMP) plays a pivotal role in numerous cellular processes, making it a potential therapeutic target. Upon activation by ligands of G-protein-coupled receptors, cAMP serves as a second messenger, initiating signaling cascades *via* protein kinase A, which in turn activates cAMP response element-binding protein [15]. This transcription factor possesses response elements found in several genes implicated in the pathogenesis of psoriasis, including IL-2, IL-6, IL-10, and TNF-α [16]. Intracellular cAMP is regulated by phosphodiesterase (PDE) [17], making PDE an attractive target for psoriasis treatment. Inhibitors of PDE can indirectly suppress the expression of inflammatory cytokines, thereby contributing to the therapeutic management of psoriasis. Given the role of PDE4 in cytokine regulation, its inhibition represents a promising therapeutic target. Roflumilast is a selective PDE inhibitor previously approved for the treatment of chronic obstructive pulmonary disease and is under investigation for the treatment of psoriasis [18].

We conducted this systematic review and meta-analysis to evaluate the current evidence on the effectiveness and safety of roflumilast for psoriasis.

## 2 METHODS

This study was conducted in accordance with the Preferred Reporting Items for Systematic Reviews and Meta-Analyses guidelines [19] and the recommendations of the Cochrane Handbook for Systematic Reviews of Interventions [20]. This study was registered with PROSPERO (CRD42024529658). The review protocol is publicly available *via* PROSPERO.

### 2.1 Database Searching

We searched the Cochrane Central Register of Controlled Trials, PubMed, Scopus, and Web of Science databases for relevant articles up to March 2024. The following search terms were used in combination with the Boolean operator: (Roflumilast OR "daliresp") AND (Psoriasis OR "psoriasis vulgaris" OR "Plaque psoriasis" OR "Guttate psoriasis" OR "Pustular psoriasis" OR "Inverse psoriasis" OR "Erythrodermic psoriasis" OR "Pustulosis of Palms and Soles" OR "Pustulosis Palmaris et Plantaris" OR "Palmoplantaris Pustulosis" OR "Pustular Psoriasis of Palms and Soles”). No time constraints were applied to the search.

### 2.2 Inclusion and Exclusion Criteria

#### 2.2.1 Inclusion Criteria

For quantitative assessment, we included all randomized controlled trials (RCTs) comparing roflumilast against placebo in patients ≥2 years with a clinical diagnosis of psoriasis. RCTs measuring severity and improvement indices were included in the study.

#### 2.2.2 Exclusion Criteria

Articles with designs other than RCTs, such as reviews, letters to editors, abstracts, opinions, and nonhuman studies, were excluded from meta-analysis. Trials that involved drugs other than roflumilast, additional interventional drugs, other comparators instead of placebo, or were not published in English were also excluded.

### 2.3 Study Selection and Data Extraction

Search results were exported to EndNote (version 20) [21], where duplicates were screened and removed. Rayyan software [22] was then used for the initial review of titles and abstracts by two independent authors to assess eligibility. For variables with missing or unclear information (*e.g.*, participant demographics or intervention details), we assumed uniformity across study arms where not specified, and incomplete outcome data were excluded from the meta-analysis to ensure robustness.

Any disagreements were resolved through discussion, with a third reviewer making the final decision if consensus was not reached. Full-text screening was conducted for eligible articles that met the inclusion criteria. References from the included studies were reviewed to identify any additional relevant studies not included in the initial search. The data from final records, including year of publication, target population, baseline characteristics, study locations, and outcomes, were manually extracted into a Google sheet by two independent reviewers.

### 2.4 Efficacy Outcomes

The primary outcomes of this study were the percentage of patients achieving Investigator’s Global Assessment (IGA) score success, defined as an IGA score of 0 (clear) or 1 (almost clear) with a 2-grade improvement, at weeks 4 and 8, as well as the percentage of patients achieving 75% or 90% reduction in the Psoriasis Area and Severity Index. Secondary outcomes were the percentages of patients achieving an Itch Numeric Rating Scale (NRS) score for psoriasis, changes in Psoriasis Symptoms Diary (PSD) Score, and the percentages of patients achieving intertriginous area IGA success.

### 2.5 Safety Outcomes

Adverse events and serious adverse events, whether treatment-related or not, were assessed. Any events reported in published clinical trials (*e.g.*, headache, diarrhea, itching, *etc*.) were considered important.

### 2.6 Risk of Bias Assessment

The risk of bias in the included clinical trials was assessed using the Cochrane Risk of Bias tool (RoB1) for interventional studies, as outlined in the official guidelines. This tool evaluates selection bias, allocation concealment, blinding of participants and personnel, blinding of outcome assessors, missing outcome data, selective reporting of outcomes, and other potential sources of bias. A high risk of bias was identified in Snape (2016) due to inadequate blinding, while an unclear risk was noted in NCT03392168 regarding allocation concealment.

### 2.7 Statistical Analysis

A meta-analysis was conducted using Review Manager (RevMan, version 5.4) [23] on the extracted outcome data available in at least three of the included studies. Similar data extraction and analysis methods have been employed in other systematic reviews, such as a study evaluating the efficacy of duloxetine for post-spine surgery pain [34]. Missing summary statistics were addressed by contacting study authors where feasible; otherwise, available data were used without imputation. Data conversions, such as transforming standard errors to standard deviations, were performed following Cochrane guidelines when necessary. Publication bias was assessed visually using funnel plots for outcomes with sufficient studies (≥10) and quantitatively using Egger’s test where applicable, in accordance with Cochrane recommendations.

Mean difference (MD) with a 95% confidence interval (CI) was used as the effect size for continuous outcomes, or standardized MD (SMD) when different outcome reporting scales were applied. For dichotomous outcomes, event frequencies and total patient numbers were pooled as a risk ratio (RR) with 95% CI. To account for inter-study heterogeneity and obtain a more conservative effect estimate, a random-effects model was adopted instead of a fixed-effects model. Heterogeneity was assessed using the chi-square test (*P* ≤ 0.1) and the I² statistic (>40%). Greater emphasis was placed on the I² statistic, as it is more reliable than the chi-square test when the number of included studies is small. To address heterogeneity, sensitivity analysis was performed.

### 2.8 Certainty Assessment

The certainty of evidence for primary outcomes (IGA and PASI) was assessed using the GRADE approach, considering risk of bias, inconsistency, indirectness, imprecision, and publication bias. Evidence was rated as high, moderate, low, or very low.

## 3 RESULTS

### 3.1 Study Selection

A systematic literature search yielded 343 potentially relevant records from PubMed, Cochrane Central Register of Controlled Trials, Scopus, and Web of Science. EndNote was used to remove 138 duplicates, leaving 205 articles for title and abstract screening. Of these, 50 articles met the eligibility criteria for our research question. Full-text assessment was then conducted, resulting in the exclusion of 45 records for various reasons. Therefore, five studies Lebwohl (2020) [24], Lebwohl (2022) [25], Kircik (2023) [26], Snape (2016) [27], and NCT03392168) were included in the qualitative synthesis, while four of them were included in the meta-analysis. The search process, as well as the number of included and excluded studies, is shown in Fig. (**1**). The 45 excluded records were primarily due to non-RCT designs (*n*=20), use of comparators other than placebo (*n*=15), or non-English publications (*n*=10). A detailed list of excluded studies and reasons is provided in the Supplementary File.

### 3.2 Characteristics of the Studies

All included RCTs evaluated the efficacy and safety of roflumilast compared to placebo/vehicle in chronic plaque psoriasis patients, published between 2016 and 2023. The analysis included four RCTs [24-26], with a total of 1609 patients. Lebwohl (2020) [24] consisted of two groups: one receiving roflumilast 0.3% cream and the other receiving roflumilast 0.15% cream. Lebwohl (2022) [25] consisted of two RCTs (DERMIS-1 and DERMIS-2), which had different sample sizes but with the same intervention. All trials were multicenter, double-blinded RCTs. Lebwohl (2020) [24], Kircik (2023) [26], and NCT03392168 were phase II trials, while Lebwohl (2022) [25] was also a phase II trial. In Lebwohl (2020) [24], some patients entered an open-label extension study for 12 months after week 12. Interventional groups were treated with different doses of roflumilast cream or foam (0.15%, 0.3%, 0.5%), applied once daily to affected areas, while the comparator group received a vehicle (placebo) cream once daily. A detailed summary of the included RCTs and the participants’ baseline characteristics is shown in Tables **1** and **2**.

### 3.3 Risk of Bias and Quality Assessment

We evaluated the risk of bias in the RCTs using the RoB1 tool. This assessment revealed that two studies were at low risk of bias, two were rated as unclear, and one was deemed to have a high risk of bias. DERMIS 1 and DERMIS 2, as reported in Lebwohl (2022), were categorized as low risk since they demonstrated minimal bias across all domains assessed by RoB1. However, Snape (2016) [27] and NCT03392168 were rated as having an unclear risk of bias due to insufficient reporting on randomization and allocation concealment. Lebwohl's study was flagged as high risk due to concerns about potential conflicts of interest among the authors (Figs. **2** and **3**).

### 3.4 Primary Outcomes

Success on the IGA scale, defined as the percentage of patients achieving an IGA status of clear (score of 0) or almost clear (score of 1) with at least a 2-grade improvement from baseline at weeks 4 and 8, was assessed in three of the included studies. The IGA was evaluated using a 5-point scale ranging from 0 (clear) to 4 (severe). Meta-analysis results revealed that roflumilast significantly improved IGA success rates compared to the control group (RR = 3.48, 95% CI [2.04–5.92], *P <* 0.00001 at week 4; RR = 4.02, 95% CI [3.17–5.^11^], *P <* 0.00001 at week 8). Low heterogeneity was observed at week 4 (I² = 38%) and week 8 (I² = 5%) (Figs. **4** and **5**).

Using the GRADE approach, the certainty of evidence for IGA and PASI outcomes was rated as moderate, primarily due to the limited number of studies and some heterogeneity, but supported by a low risk of bias in key trials and precise effect estimates.

The severity index was assessed using two different scores. Lebwohl (2020) and Lebwohl (2022) utilized the Psoriasis Area and Severity Index score, whereas Kircik (2023) adopted the Palmoplantar Psoriasis Severity Index score. The percentage of patients achieving a 75% reduction in the severity index was evaluated at weeks 4 and 8. Due to the significant unexplained heterogeneity in the severity index at both weeks 4 (*P =* 0.0001, I² = 83%) and 8 (*P =* 0.009, I² = 70%), subgroup analysis was performed. Results showed that the overall relative risk (RR) between roflumilast and placebo favors roflumilast (RR = 2.72, 95% CI [1.18 to 6.^28^], *P <* 0.00001) and (RR = 3.41, 95% CI [2.19 to 5.^32^], *P <* 0.00001) at weeks 4 and 8, respectively. After applying subgroup analysis, pooled studies were homogenous, with I² = 0% and 4% at week 4 and I² = 9% and 0% at week 8 (Figs. **6** and **7**).

The percentage of patients achieving a 90% reduction in the severity index at weeks 4 and 8 was also assessed. The forest plot showed high heterogeneity (*P =* 0.01, I² = 70%) at week 8, but when a subgroup analysis was conducted, the pooled results of heterogeneity decreased significantly (I² = 0% and 33%, with *P =* 0.85 and *P =* 0.22, respectively) under the random-effects model. Pooled studies were homogenous at week 4 (*P =* 0.28, I² = 21%). Pooled analysis showed that roflumilast significantly reduced the severity index compared to placebo at weeks 4 (RR = 4.27, 95% CI [1.84 to 9.89], *P =* 0.0007) and 8 (RR = 5.06, 95% CI [2.24 to 11.^4^], *P <* 0.0001; Figs. **8** and **9**).

### 3.5 Secondary Outcomes

Roflumilast was significantly associated with an improvement in the PSD score at both week 2 (MD: -14.11, 95% CI [-19.74, -8.48], *P ˂* 0.00001) and week 6 (MD: -22.46, 95% CI [-29.93, -15.0], *P <* 0.00001), as shown in Fig. (**9**). Additionally, roflumilast demonstrated a significant improvement in the Worst Itch NRS score at week 4 (RR = 2.95, 95% CI [2.20, 3.0^4^], *P ˂* 0.00001) and week 8 (RR = 1.95, 95% CI [1.51, 2.53], *P ˂* 0.00001), as shown in the supplementary figures.

The meta-analysis results revealed that roflumilast showed a significant improvement in IGA score of 0 or 1 among patients with a baseline I-IGA score of ≥2 at both weeks 4 (RR = 1.97, 95% CI [1.33, 2.93], *P =* 0.0008) and 8 (RR = 3.83, 95% CI [2.25, 6.51], *P ˂* 0.00001). Moreover, the results also favored roflumilast over placebo in improving the IGA score of 0 or 1 among patients with I-IGA ≥2 and at least a 2-grade improvement score from baseline at week 4 (RR = 2.30, 95% CI [1.64, 3.^21^], *P ˂* 0.00001) and week 8 (RR = 2.94, 95% CI [1.88, 4.59], *P ˂* 0.00001), as shown in the supplementary figures.

Pooled studies showed unexplained heterogeneity. Subgroup analysis was conducted and completely resolved the heterogeneity in the PSD outcome at both week 2 (I² = 62% to 0%) and week 6 (I² = 72% to 0%). Additionally, the heterogeneity in IGA score of 0 or 1 among patients with baseline I-IGA score ≥2 at week 8 was resolved by subgroup analysis (I² = 89% to 0%). At week 4, sensitivity analysis resolved the heterogeneity in the IGA score of 0 or 1 among patients with baseline intertriginous-area IGA score of ≥2 with a 2-grade improvement, as well as in the Worst Itch NRS outcome, as shown in the supplementary figures. The remaining endpoints assessed were not resolved by either subgroup or sensitivity analysis.

### 3.6 Safety Outcomes

Regarding side effects, no statistically significant difference was found between roflumilast and placebo in the development of any treatment-emerging adverse events (TEAEs), treatment-related TEAEs, serious adverse events, adverse events leading to study withdrawal, or in the incidence of psoriasis, nasopharyngitis, or hypertension. However, the meta-analysis showed that roflumilast was associated with a higher risk of developing diarrhea (RR = 7.32, 95% CI [1.39, 38.^5^], *P =* 0.02) and a lower risk of pain at the site of application (RR = 0.36, 95% CI [0.16, 0.81], *P =* 0.01) compared to placebo. Pooled studies were homogeneous in all assessed outcomes.

A summary of the safety outcome measures is illustrated in Table **3**, with the forest plot figures in the supplementary materials.

### 3.7 Reporting Bias Assessment

Due to the limited number of studies included (*n*=5), funnel plots and Egger’s test were not feasible for assessing publication bias, as fewer than 10 studies reduce the reliability of these methods. No evidence of selective reporting was identified within the included studies.

## 4 DISCUSSION

In this systematic review and meta-analysis, we aimed to evaluate the current evidence regarding the effectiveness and safety of roflumilast, a PDE inhibitor, in the treatment of psoriasis. In addition to the review, we have performed a comprehensive meta-analysis to estimate the pooled effect of different doses of roflumilast compared to placebo. This pooled effect was estimated from four RCTs. Furthermore, we evaluated the drug’s tolerability through an extensive pooled safety analysis from five RCTs. To our knowledge, this is the first meta-analysis evaluating roflumilast for psoriasis.

Psoriasis is a chronic, debilitating autoimmune disease that affects patients’ quality of life [28]. It is a dermatological disease well-known for its three cardinal features: erythema, thickening, and scaling [29]. Ranging from well-demarcated erythematous lamellar silver scales on large areas of the body [30] to severe erythroderma [30], plaque psoriasis is the most common type of psoriasis, affecting 80%-90% of psoriasis patients [31]. Although corticosteroids are the cornerstone of psoriasis treatment, their long-term use can result in serious adverse events [32]. Roflumilast is a phosphodiesterase inhibitor that regulates the levels of cAMP involved in the activation of multiple proinflammatory cytokines in the pathogenesis of psoriasis, such as IL-2, IL-6, and TNF-α. It has 200 times the potency and affinity of other PDE inhibitors [33]. This explains the rationale for using roflumilast in the treatment of psoriasis, which is reflected in our pooled effect estimates.

The combined findings from our meta-analysis indicate that roflumilast effectively reduced the proportion of patients achieving an IGA status of clear (0) or almost clear (1) with a 2-grade improvement from baseline at both weeks 4 and 8 compared to placebo. These results were statistically significant and exhibited negligible heterogeneity. Additionally, roflumilast increased the percentage of patients achieving a 75% reduction in the Psoriasis Area and Severity Index at both weeks 4 and 8 compared to placebo [34]. However, significant unexplained variability was observed at week 4, which was resolved through subgroup analysis by grouping the results of Lebwohl (2020; 0.15% and 0.3%). Despite this, significant heterogeneity persisted at week 8.

Furthermore, roflumilast demonstrated a significant increase in the percentage of patients achieving a 90% reduction in the Psoriasis Area and Severity Index at both weeks 4 and 8, although unexplained significant heterogeneity was present. Subgroup analysis, incorporating the results of Lebwohl (2020; 0.15% and 0.3%), resolved this heterogeneity in both weeks. Regarding secondary efficacy outcomes, roflumilast reduced mean PSD and NRS scores at weeks 2, 4, and 6. In terms of safety, aside from diarrhea, roflumilast exhibited no statistically significant difference compared to placebo, and the results were consistent.

The clinical relevance of roflumilast can be better understood when compared to other novel topical agents, such as Tapinarof and Crisaborole. Tapinarof, an aryl hydrocarbon receptor (AhR) agonist, has shown comparable or slightly superior efficacy in clearing plaques but may be associated with more frequent application site reactions and folliculitis. Crisaborole, a topical PDE-4 inhibitor primarily used for atopic dermatitis, has limited efficacy in plaque psoriasis and is generally considered less potent than roflumilast. Notably, roflumilast’s once-daily dosing, favorable safety profile, and non-steroidal formulation provide important advantages, particularly for long-term disease control and patient adherence. These attributes position roflumilast as a competitive alternative in the landscape of topical treatments, especially in patients for whom steroid-sparing regimens are preferred.

## 5 STUDY LIMITATIONS

This study has several limitations. First, the number of clinical trials currently available to assess the effectiveness and safety of roflumilast is limited, and more comprehensive, well-designed RCTs are needed to obtain conclusive evidence. Second, the meta-analysis of some outcomes showed heterogeneity that could not be resolved. For certain outcomes, marked heterogeneity was observed, which could have been addressed through sensitivity or subgroup analyses; however, it could not be fully explained despite all trials sharing similar patient selection criteria, methodologies, and outcome assessment methods. Additionally, the review process may have been limited by the reliance on four major databases, potentially missing studies from other sources, and by the manual data extraction process, which could introduce errors despite independent verification by two reviewers. The heterogeneity observed in PASI 75 outcomes may also reflect differences in baseline disease severity across trials.

## CONCLUSION

This meta-analysis represents the first examination of the efficacy and safety profile of roflumilast in the management of psoriasis. The findings indicate that roflumilast demonstrates potential effectiveness and tolerability in psoriasis treatment. However, further rigorous clinical trials are warranted to substantiate these findings.

## Figures and Tables

**Fig. (1):**
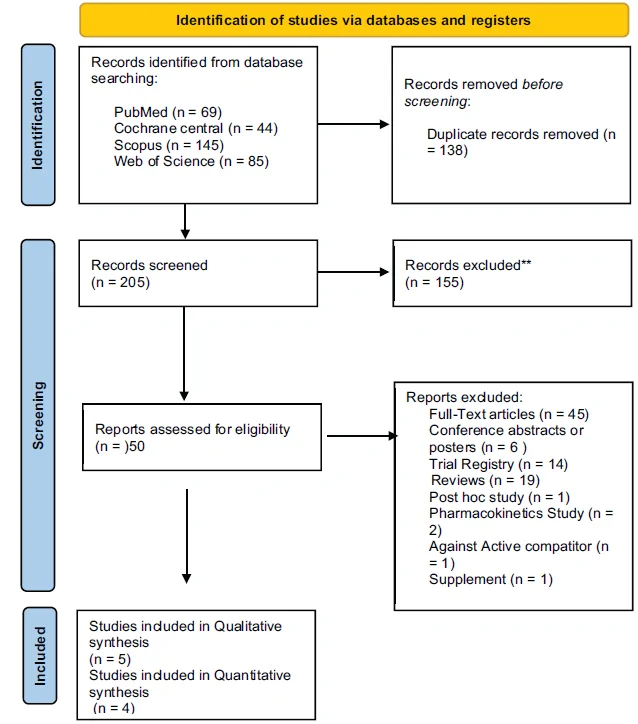
Showing PRISMA flow diagram.

**Fig. (2):**
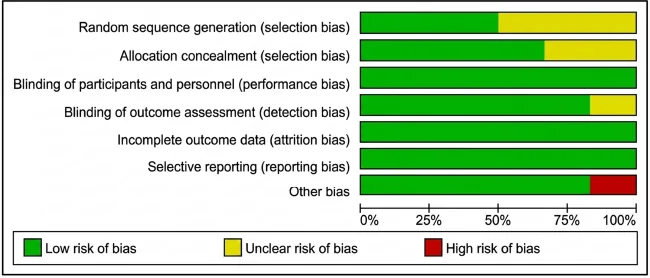
Showing risk of bias graph.

**Fig. (3):**
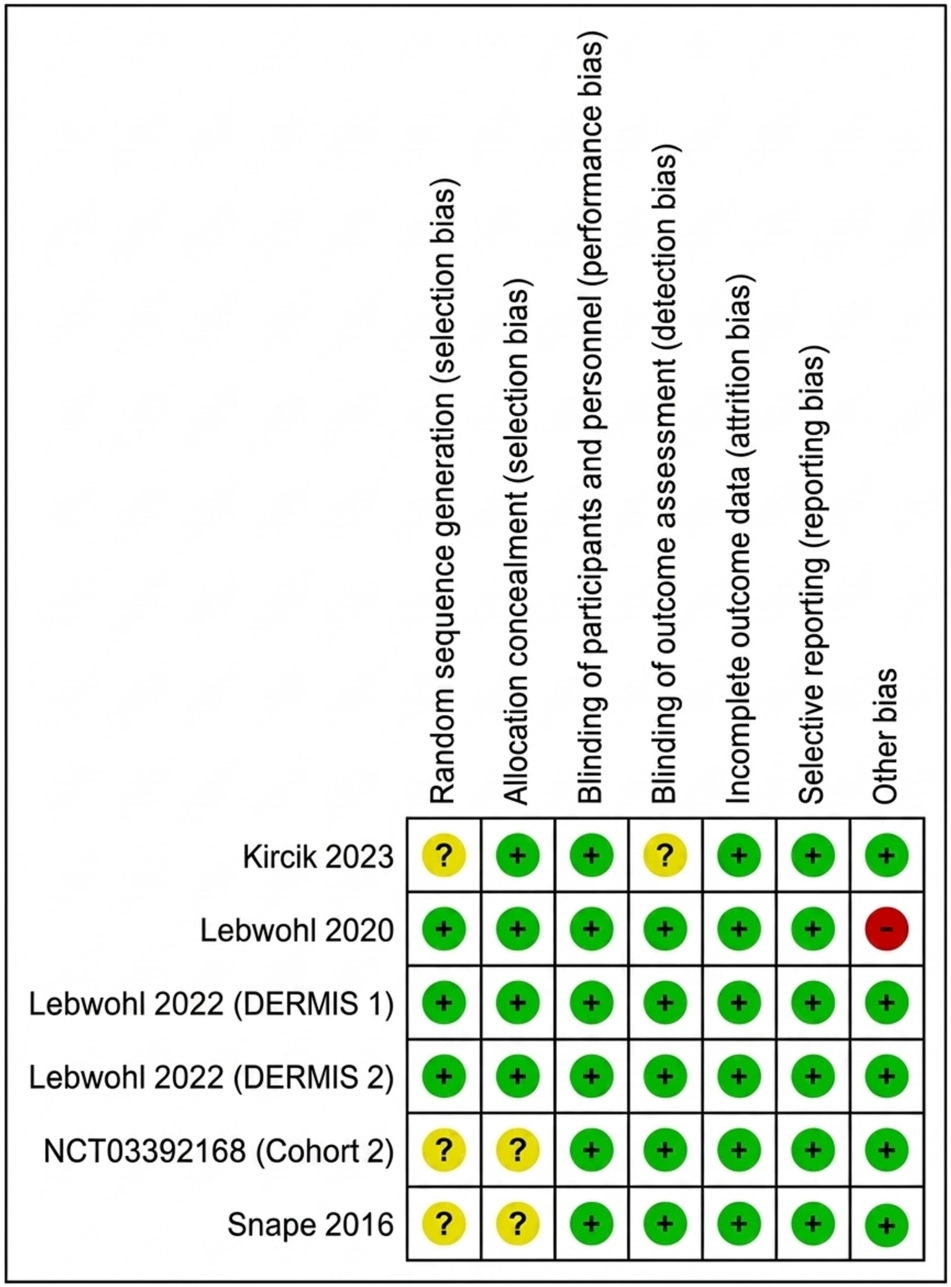
Showing risk of bias summary.

**Fig. (4):**
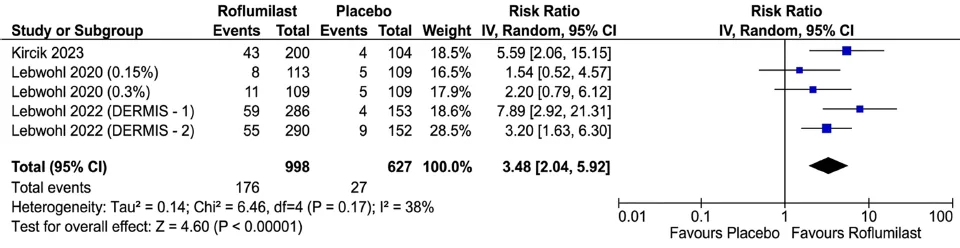
Showing forest plot of IGA success at week 4.

**Fig. (5):**
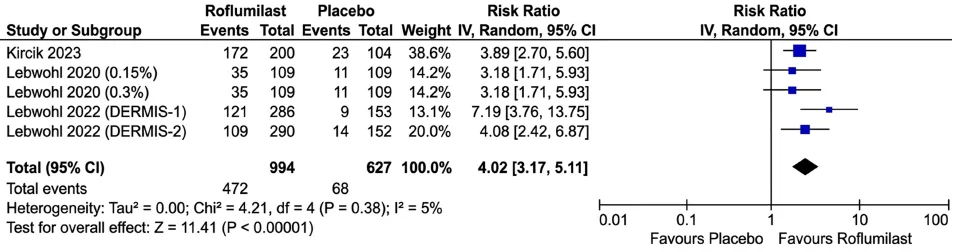
Showing forest plot of IGA success at week 8.

**Fig. (6):**
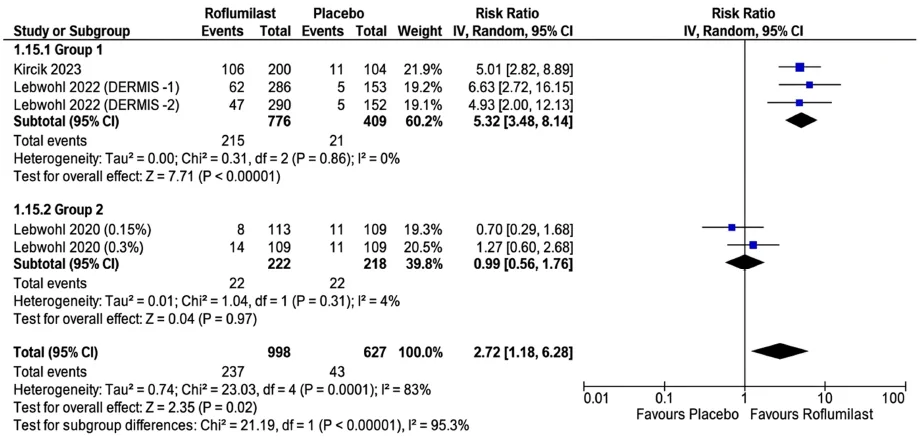
Showing forest plot of PASI 75 % change at week 4.

**Fig. (7):**
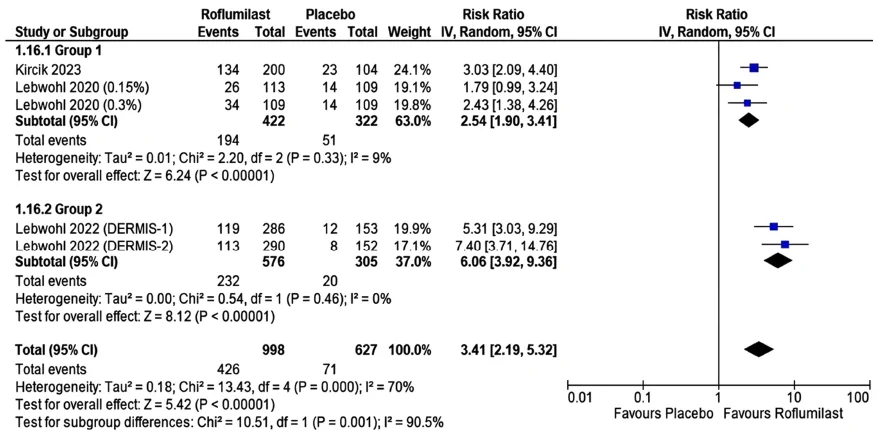
Showing forest plot of PASI 75 % change at week 8.

**Fig. (8):**
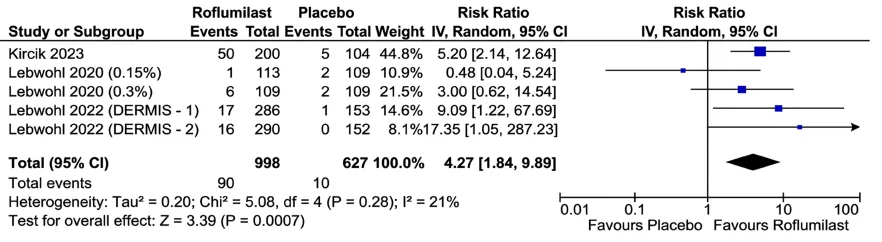
Showing forest plot of PASI 90% change at week 4.

**Fig. (9):**
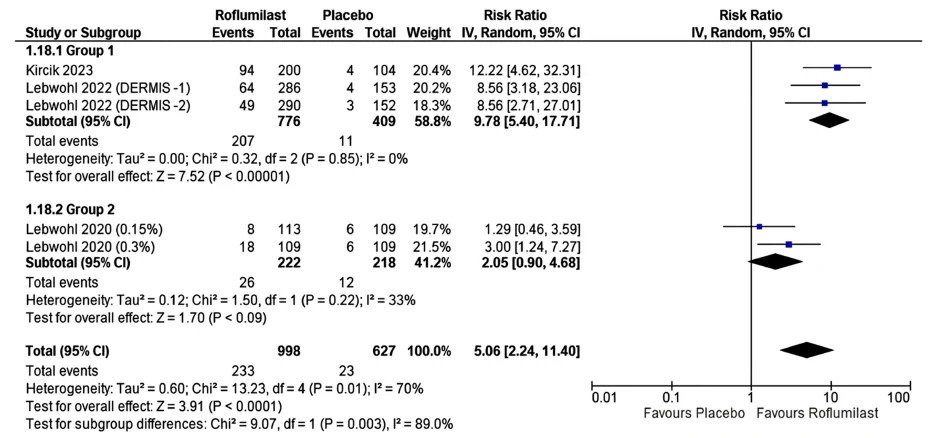
Showing forest plot of PASI 90% change at week 4.

**Table 1:** Showing summary of included studies.

**Study ID**		**Study Design**	**Study Duration (Weeks)**	**Interventions**	**Control**	**Sample Size**		**Efficacy Outcomes Assessed**
						**Intervention**	**Control**	
Lebwohl 2020		RCT	12	G1: roflumilast 0.3% cream, G2: roflumilast 0.15% cream	Vehicle	G1: 109 G2: 113	109	- Change in the PASI score - Change in PSD score - NRS response among patients with baseline WI-NRS score ≥ 6. - IGA status of clear or almost clear
Leb-wohl 2022	DERMIS-1	RCT	8	Roflumilast 0.3% cream	Vehicle	286	153	- Percentage of Patients Achieving IGA Success - Achievement of 75% & 90% reduction in PASI score - Percentage pf patients achieving WI-NRS success - Change from baseline PSD score - IGA status of clear or almost clear
	DERMIS-2					290	152	
Kircik 2023		RCT	8 + 1 follow up	Roflumilast foam 0.3%	Vehicle	200	114	- Percentage of patients S-IGA success - Percentage of patients achieving a 4-point SI-NRS - Percentage of patients achieving B-IGA success - Change in PSD and PSSI score - Percentage of patients achieving a 4-point reduction in WI-NRS - Change from baseline in DLQI
Snape 2016			3 + 2 follow up	G1: 0.005% calcipotriol cream; G2: 0.1% betamethasone valerate G3: 0.5% roflumilast, G4: 5% TAK-084 , G5: 0.5% TAK-084	Vehicle	15		- Change from baseline in thickness of the psoriatic skin infiltrate by ultrasonography - Change in clinical severity of the psoriatic lesions within each test area by comparing photos
NCT03392168	RCT	4	G1: roflumilast cream 0.5%, G2: roflumilast cream 0.15%	Vehicle	G1: 28 G2: 26	29		- Percent Change from Baseline in target Plaque Severity Score x Target Plaque Area - Percent Change from Baseline in Total Plaque Severity Score - Percent Change From Baseline in target plaque area

**Note:** * for 12 weeks then placebo patients were switched to open-label roflumilast through week 24.

**Abbreviations:** PASI: psoriasis area and severity index; PSD: Psoriasis Symptom Diary; WI-NRS: Worst Itch Numeric Rating Scale; DLQI: dermatology life quality index; S-IGA: scalp Investigator’s Global Assessment; SI-NRS: Scalp Itch Numeric Rating Scale; B-IGA: body Investigator’s Global Assessment

**Table 2:** Showing baseline characteristics of included studies.

**Study ID**	**Study Arms**	**Sample Size**	**Age *mean ± SD***	**Sex, males *n (%)***	**BMI *mean ± SD***	**Percent of Body-Surface Area Affected by Psoriasis** **Mean±SD**	**Psoriasis Symptom Diary Total Score**	**PASI** **Mean±SD**	**Intertriginous Area Involvement n(%)**
Lebwohl 2020	Roflumilast 0.3% cream	109	51.7 ± 14.1	56 (51%)	NR	6.3± 4	68.9± 41.2	7.7± 3.6	16(15)
	Roflumilast 0.15% cream Placebo	113 109	54.4 ± 14.2 55.5 ± 13.5	62 (55 %) 56 (61%)	NR	6.4± 3.9 6.4± 3.6	69.6± 46.2 75.1± 42.6	8± 3.9 7.6± 3.1	18(16) 17(16)
Kircik 2023	Roflumilast 0.3% cream	200	45.2±14.22	96(48%)	NR	8 ± 5.63	NR	7.18 ± 4.29	
	Placebo	104	45 ±15.76	47(47%)	NR	7.6 ± 5.62	NR	6.78 ± 4.38	
Lebwohl 2022 (DERMIS -1)	Roflumilast 0.3% cream	286	47.6± 14.1	189(66.1)	32± 9.5	6.3± 4.4	72.1± 42.75	6.3± 3.15	68(23.8)
	Placebo	154	48.7± 15.8	96(62.7)	31.9 ±7.9	7.4± 4.8	73.4± 41.29	6.8± 3.7	33(21.6)
Lebwohl 2022 (DERMIS -2)	Roflumilast 0.3% cream	290	46.9±15.07	176(60.7)	31± 7	7.1± 4.8	69.3± 40.66	6.5±3.22	56(19.3)
	Placebo	152	47.1±14.07	100(65.8)	33.5± 13.7	7.7± 5.1	77.4± 41.24	7±3.52	32(21.1)
Snape 2016 NCT03392168 (Cohort 2 )	"0.005% calcipotriol cream / 0.1% betamethasone valerate / 0.5% roflumilast / 0.5% and 5% TAK-084 / placebo	15	49.1±12.41	12(80)	27.6±3.32				
	Roflumilast 0.5% cream Roflumilast 0.15% cream Placebo	28 29 26	50.8±15.76 54.8± 13.5 47±13.97	16(57.1) 19(73.1) 17(58.6)					

**Table 3:** A summary for the safety outcome measures.

Outcome Measure-Period	Total No. of Studies	Total No. of Participants	Treatment Effect Estimate (95% Confidence Interval)	*P*-value	Heterogeneity
Any TEAEs	5	1598	1.03 [0.84, 1.25]	*P =* 0.78	Chi² = 8.26, (*P =* 0.22); I² = 27%
Treatment-related TEAEs	4	1509	0.77 [0.49, 1.22]	*P =* 0.27	Chi² = 3.21, (*P =* 0.52); I² =0%
Serious AEs	4	1509	0.6 [0.19, 1.96]	*P =* 0.4	Chi² = 1.21, (*P =* 0.88); I² = 0%
AEs causing permanent discontinuation of study drug	4	1509	0.69 [0.28, 1.68]	*P =* 0.41	Chi² = 3.42, (*P =* 0.49); I² = 0%
Psoriasis	3	1183	0.29 [0.06, 1.43]	*P =* 0.13	Chi² = 1.86, (*P =* 0.39); I² = 0%
Nasopharyngitis	3	1207	0.83 [0.38, 1.82]	*P =* 0.65	Chi² = 0.2, (*P =* 0.98); I² = 0%
Hypertension	4	1509	0.94 [0.4, 2.2]	*P =* 0.89	Chi² = 3.67, (*P =* 0.45); I² = 0%
Diarrhea	3	1183	7.32 [1.39, 38.5]	*P =* 0.02	Chi² = 0.31, (*P =* 0.85); I² = 0%
Application site pain	3	717	0.36 [0.16, 0.81]	*P =* 0.01	Chi² = 0.82, (*P =* 0.94); I² = 0%

## Data Availability

The data that support the findings of this study are available on request

## LIST OF ABBREVIATIONS

AhR: aryl hydrocarbon receptor
NRS: Numeric Rating Scale
PSD: Psoriasis Symptoms Diary

## STANDARDS OF REPORTING

PRISMA guidelines and methodology were followed.

## SUPPLEMENTARY MATERIAL

PRISMA checklist is available as supplementary material on the publisher’s website along with the published article.
