# Supplementary material for: Effectiveness and Safety of Roflumilast in the Treatment of Psoriasis: A Systematic Review and Meta-Analysis
Source: Curr Drug Saf. 2026 Feb 10;21(5):E15748863406187. doi: 10.2174/0115748863406187251031101244 (PMC13621373; doi:10.2174/0115748863406187251031101244)
Supplement: Supplementary file 1 [file CDS-21-5-E15748863406187_SD1.pdf]

# Supplementary Material

# Effectiveness and Safety of Roflumilast in the Treatment of Psoriasis: A Systematic Review and Meta-Analysis

Ali Mohammed Aleid[1,*], Houriah Yasir Nukaly[2], Salma Mohammed Alshreef[3], Mishari Tariq Alrubaiaan[4], Shahad Hamoud Alrowais[5], Mohammed Khalid Alghamdi[6], Shatha Saleh Al Ghamdi[7], Laila Mohammed Alali[8], Mohammad A. Alghamdi[9] and Mahdi Al Dhafiri[10]

*[1]Dermatology Department, Ministry of Health, King Faisal University (KFU), Saudi Arabia; Moleer Clinic, Riyadh, Saudi Arabia; [2]Medicine Program, Batterjee Medical College, Jeddah 21442, Saudi Arabia; [3]Umm Al-Qura University, 6th-year Medical Student, Saudi Arabia; [4]King Saud Bin Abdulaziz University for Health Sciences, College of Medicine, Riyadh, Saudi Arabia; [5]Princess Nourah Bint Abdulrahman University, College of Medicine, Riyadh, Saudi Arabia; [6]University of Jeddah, College of Medicine, Jeddah, Saudi Arabia; [7]King Abdulaziz University, College of Medicine, Jeddah, Saudi Arabia; [8]Jazan University, College of Medicine, Saudi Arabia; [9]Internal Medicine Department, Division of Dermatology, Faculty of Medicine, Al-Baha University, Al-Baha, Saudi Arabia; [10]Division of Dermatology, College of Medicine, King Faisal University, Al-Ahsa, Saudi Arabia*

## FOREST PLOT OF SAFETY ANALYSIS

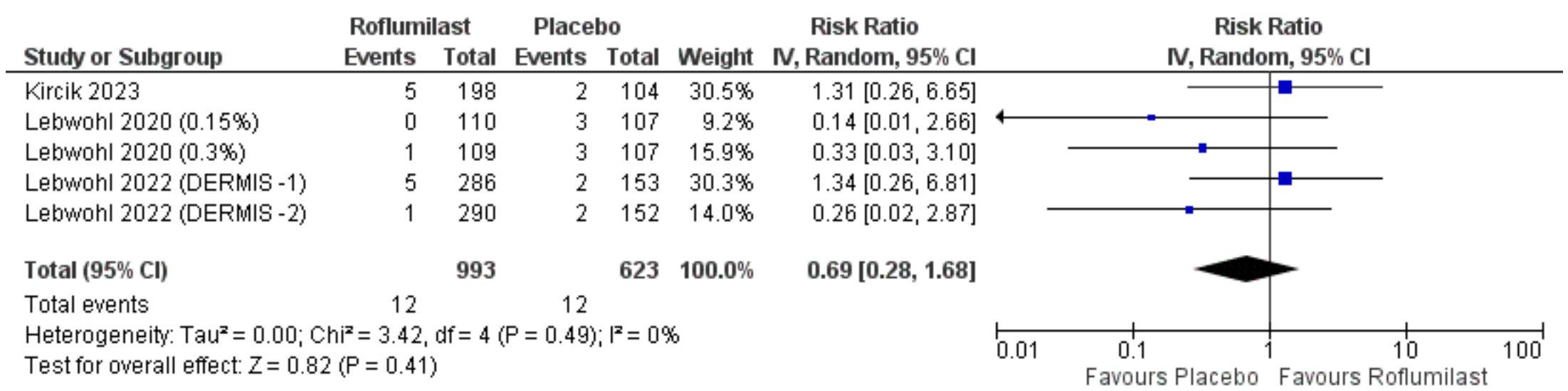


**Figure 1. Showing forest plot of AE leading to withdrawal from the study**

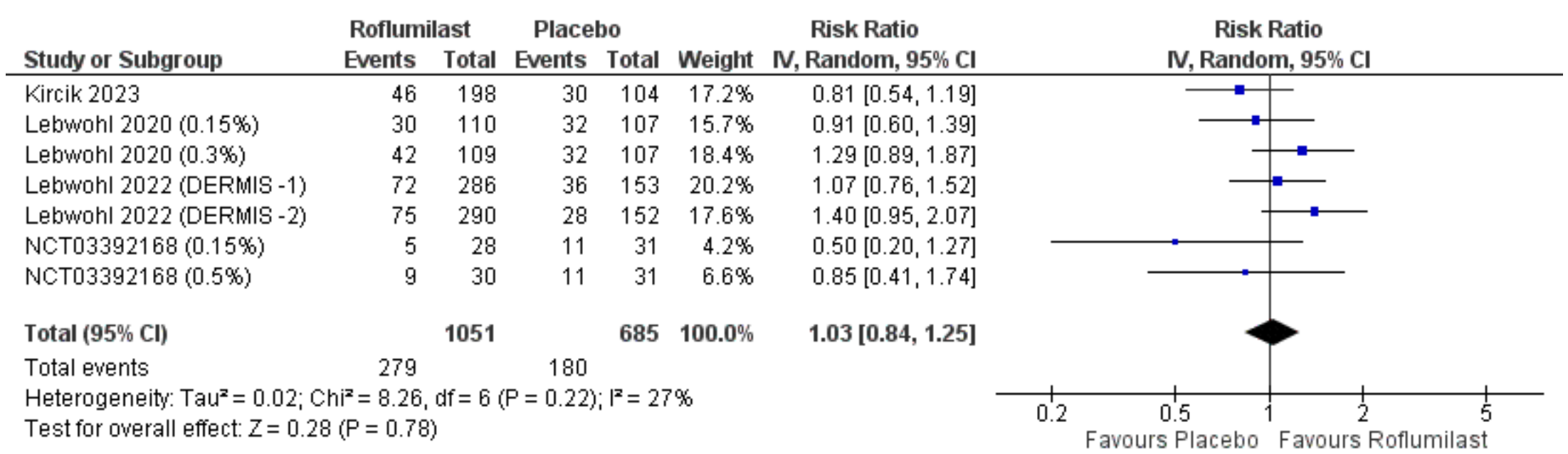


**Figure 2. Showing forest plot of any TEAEs**

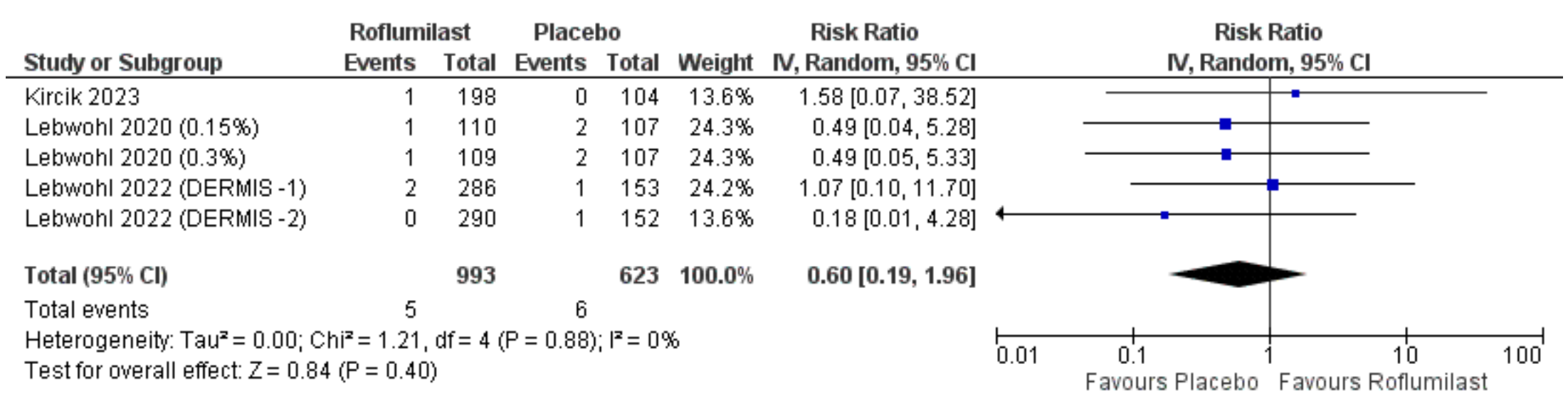


**Figure 3. Showing forest plot of serious TEAEs**

| Study or Subgroup | Roflumilast Events | Roflumilast Total | Placebo Events | Placebo Total | Weight | Risk Ratio IV, Random, 95% CI |
|---|---|---|---|---|---|---|
| Kircik 2023 | 8 | 198 | 9 | 104 | 24.8% | 0.47 [0.19, 1.17] |
| Lebwohl 2020 (0.15%) | 3 | 110 | 7 | 107 | 12.0% | 0.42 [0.11, 1.57] |
| Lebwohl 2020 (0.3%) | 7 | 109 | 7 | 107 | 20.5% | 0.98 [0.36, 2.70] |
| Lebwohl 2022 (DERMIS-1) | 7 | 286 | 3 | 153 | 11.8% | 1.25 [0.33, 4.76] |
| Lebwohl 2022 (DERMIS-2) | 16 | 290 | 8 | 152 | 30.9% | 1.05 [0.46, 2.39] |
| **Total (95% CI)** | | **993** | | **623** | **100.0%** | **0.77 [0.49, 1.22]** |
| Total events | 41 | | 34 | | | |

Heterogeneity: Tau² = 0.00; Chi² = 3.21, df = 4 (P = 0.52); I² = 0%
Test for overall effect: Z = 1.10 (P = 0.27)

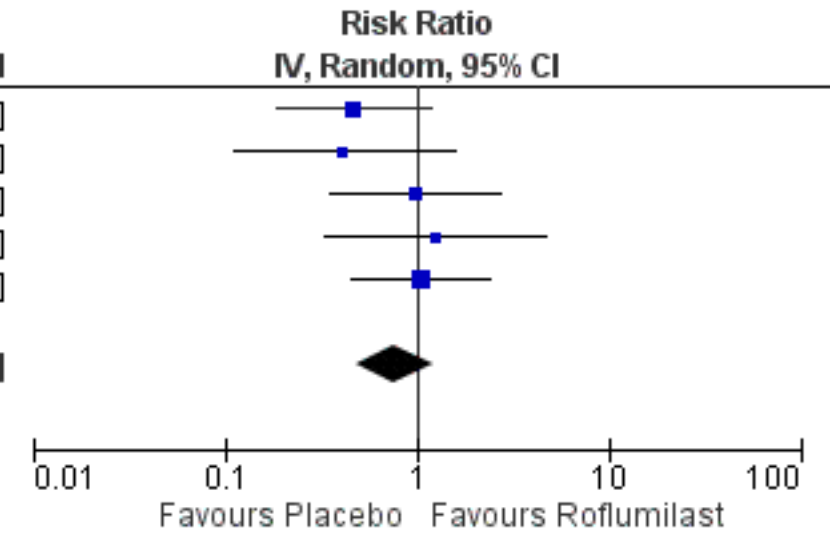


**Figure 4. Showing forest plot of treatment related TEAEs**

| Study or Subgroup | Roflumilast Events | Roflumilast Total | Placebo Events | Placebo Total | Weight | Risk Ratio IV, Random, 95% CI |
|---|---|---|---|---|---|---|
| Kircik 2023 | 3 | 198 | 0 | 104 | 31.6% | 3.69 [0.19, 70.83] |
| Lebwohl 2022 (DERMIS-1) | 10 | 286 | 0 | 153 | 34.4% | 11.27 [0.66, 191.00] |
| Lebwohl 2022 (DERMIS-2) | 8 | 290 | 0 | 152 | 34.0% | 8.94 [0.52, 153.82] |
| **Total (95% CI)** | | **774** | | **409** | **100.0%** | **7.32 [1.39, 38.50]** |
| Total events | 21 | | 0 | | | |

Heterogeneity: Tau² = 0.00; Chi² = 0.31, df = 2 (P = 0.85); I² = 0%
Test for overall effect: Z = 2.35 (P = 0.02)

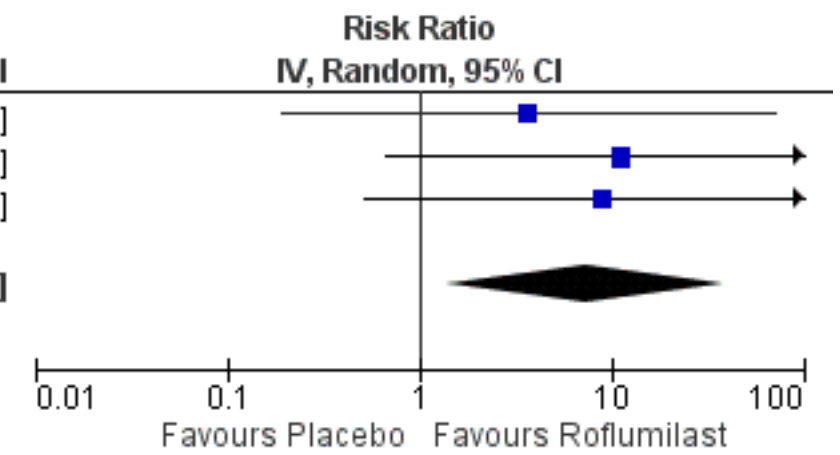


**Figure 5. Showing forest plot of Diarrhea**

| Study or Subgroup | Roflumilast Events | Roflumilast Total | Placebo Events | Placebo Total | Weight | Risk Ratio IV, Random, 95% CI |
|---|---|---|---|---|---|---|
| Kircik 2023 | 1 | 198 | 2 | 104 | 45.2% | 0.26 [0.02, 2.86] |
| Lebwohl 2022 (DERMIS-1) | 0 | 286 | 3 | 153 | 29.5% | 0.08 [0.00, 1.47] |
| Lebwohl 2022 (DERMIS-2) | 1 | 290 | 0 | 152 | 25.3% | 1.58 [0.06, 38.49] |
| **Total (95% CI)** | | **774** | | **409** | **100.0%** | **0.29 [0.06, 1.43]** |
| Total events | 2 | | 5 | | | |

Heterogeneity: Tau² = 0.00; Chi² = 1.86, df = 2 (P = 0.39); I² = 0%
Test for overall effect: Z = 1.52 (P = 0.13)

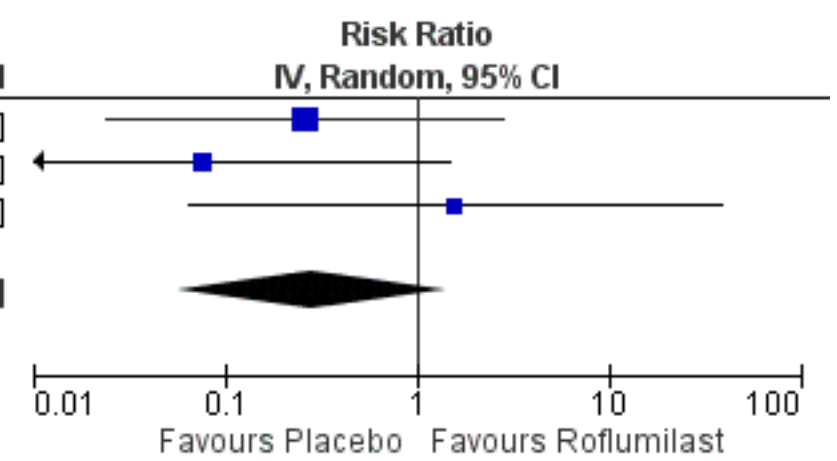


**Figure 6. Showing forest plot of Psoriasis**

| Study or Subgroup | Roflumilast Events | Roflumilast Total | Placebo Events | Placebo Total | Weight | Risk Ratio IV, Random, 95% CI |
|---|---|---|---|---|---|---|
| Lebwohl 2020 (0.15%) | 3 | 110 | 4 | 107 | 28.3% | 0.73 [0.17, 3.18] |
| Lebwohl 2020 (0.3%) | 4 | 109 | 4 | 107 | 33.2% | 0.98 [0.25, 3.82] |
| Lebwohl 2022 (DERMIS-1) | 5 | 286 | 3 | 153 | 30.5% | 0.89 [0.22, 3.68] |
| Lebwohl 2022 (DERMIS-2) | 1 | 290 | 1 | 152 | 8.0% | 0.52 [0.03, 8.32] |
| **Total (95% CI)** | | **795** | | **519** | **100.0%** | **0.83 [0.38, 1.82]** |
| Total events | 13 | | 12 | | | |

Heterogeneity: Tau² = 0.00; Chi² = 0.20, df = 3 (P = 0.98); I² = 0%
Test for overall effect: Z = 0.46 (P = 0.65)

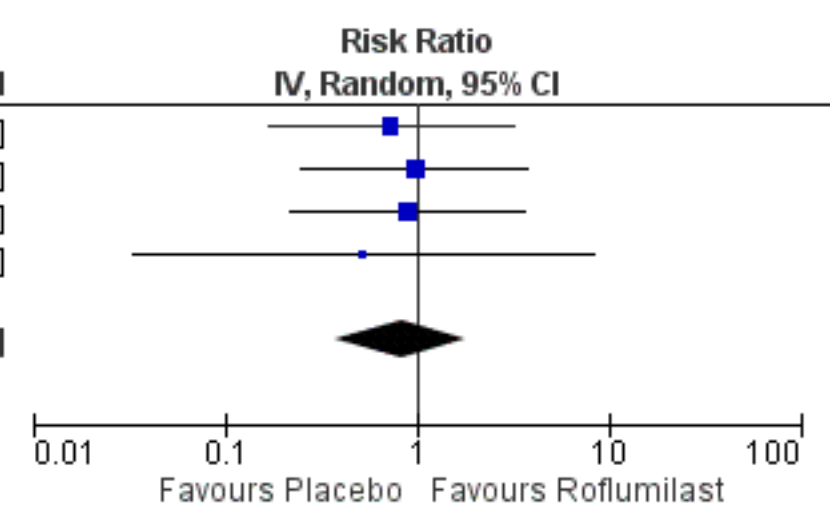


**Figure 7. Showing forest plot of Nasopharyngitis**

| Study or Subgroup | Roflumilast Events | Roflumilast Total | Placebo Events | Placebo Total | Weight | Risk Ratio IV, Random, 95% CI |
|---|---|---|---|---|---|---|
| Kircik 2023 | 3 | 198 | 1 | 104 | 14.1% | 1.58 [0.17, 14.96] |
| Lebwohl 2020 (0.15%) | 2 | 110 | 1 | 107 | 12.6% | 1.95 [0.18, 21.14] |
| Lebwohl 2020 (0.3%) | 2 | 109 | 1 | 107 | 12.6% | 1.96 [0.18, 21.33] |
| Lebwohl 2022 (DERMIS-1) | 5 | 286 | 6 | 153 | 52.3% | 0.45 [0.14, 1.44] |
| Lebwohl 2022 (DERMIS-2) | 4 | 290 | 0 | 152 | 8.4% | 4.73 [0.26, 87.31] |
| **Total (95% CI)** | | **993** | | **623** | **100.0%** | **0.94 [0.40, 2.20]** |
| Total events | 16 | | 9 | | | |

Heterogeneity: Tau² = 0.00; Chi² = 3.67, df = 4 (P = 0.45); I² = 0%
Test for overall effect: Z = 0.14 (P = 0.89)

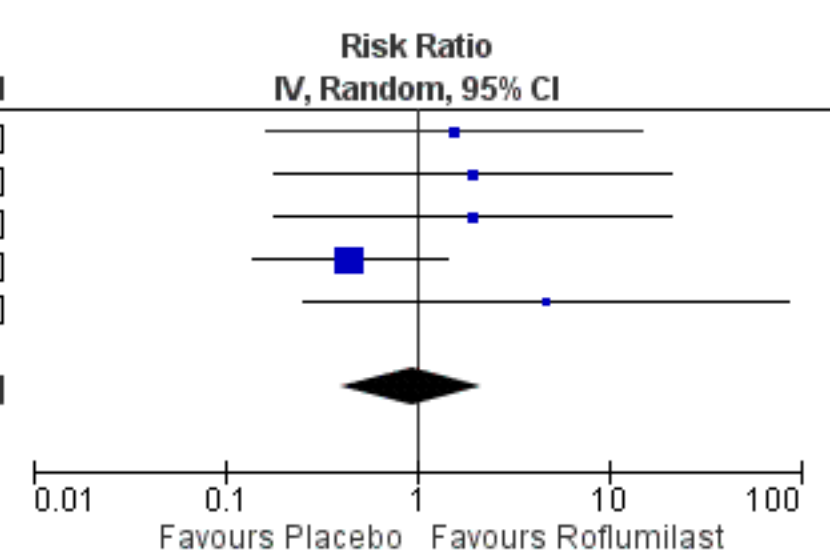


**Figure 8. Showing forest plot of Hypertension**

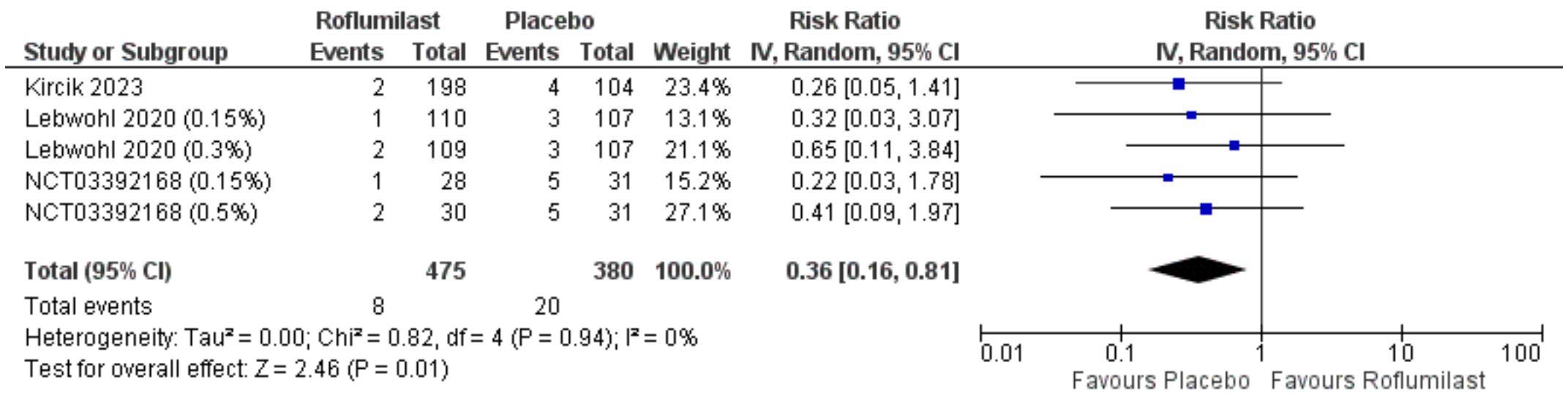


**Figure 9. Showing forest plot of application site pain**

## SECONDARY OUTCOME

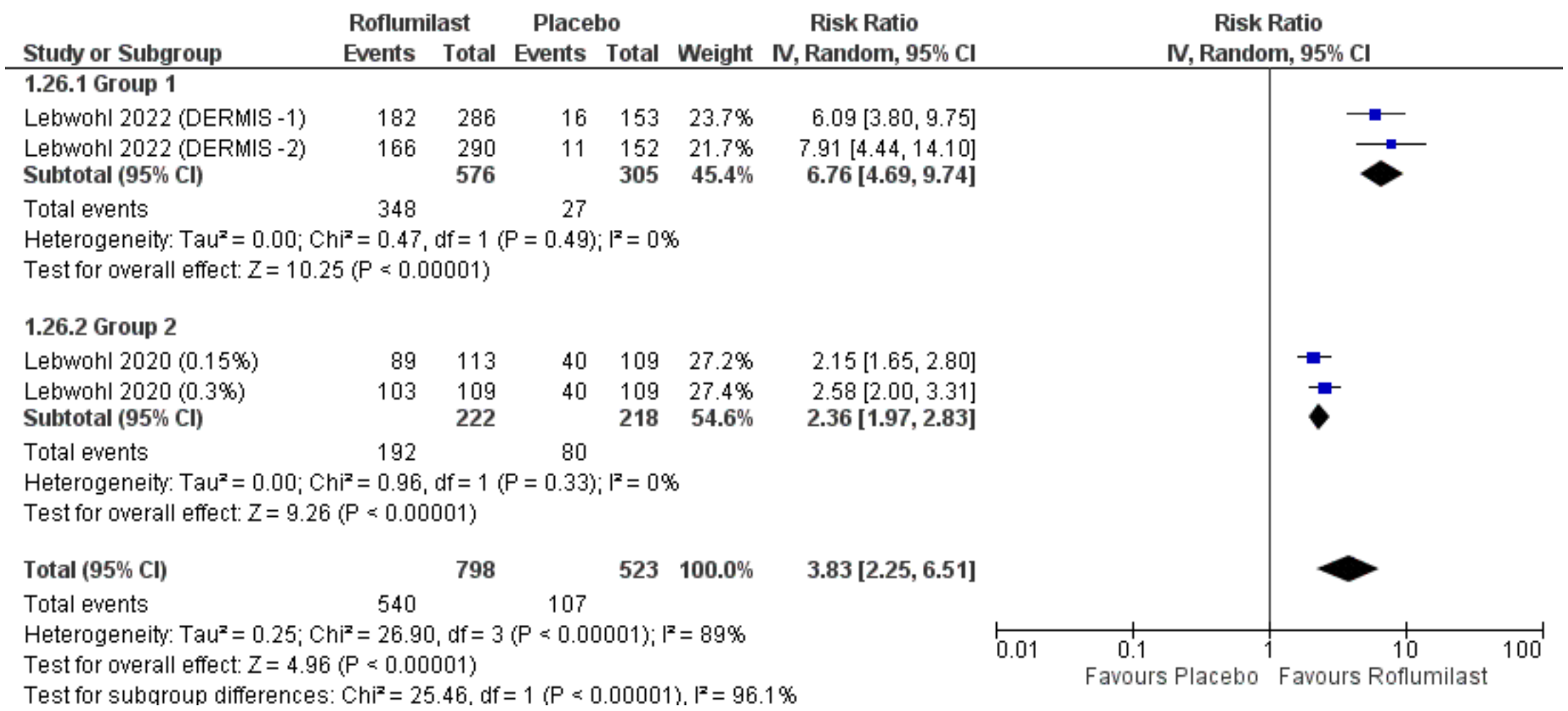


**Figure 10. Showing IGA score of 0 or 1 among patients with baseline intertriginous at week 8**

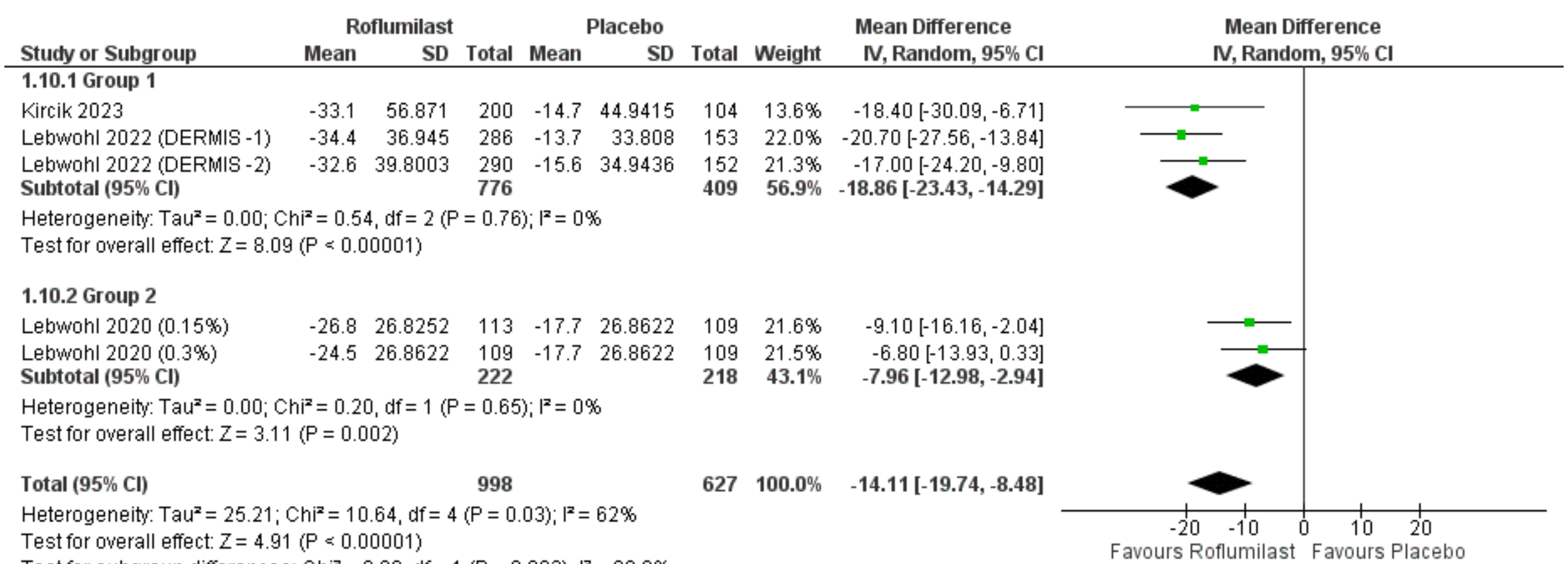


**Figure 11. Showing change of PSD score at week 2**

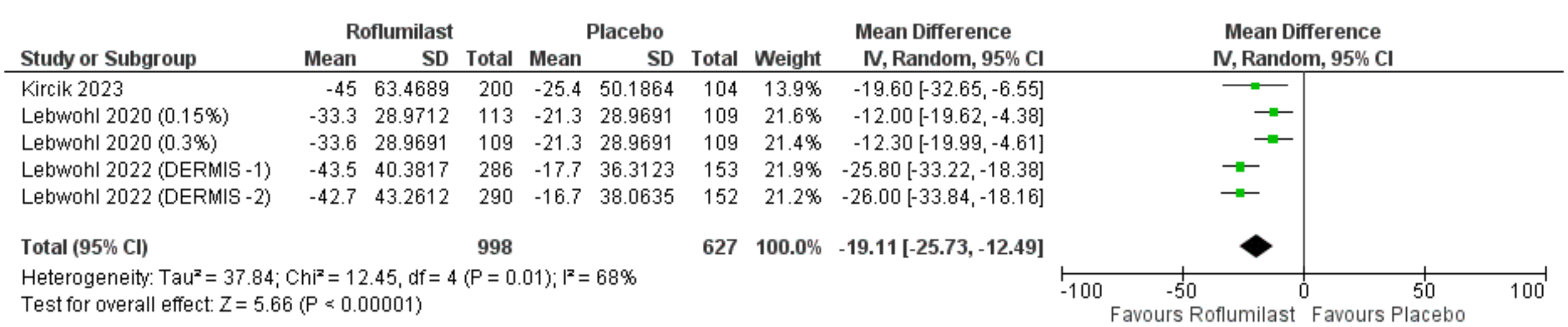


**Figure 12. Showing change of PSD score at week 4**

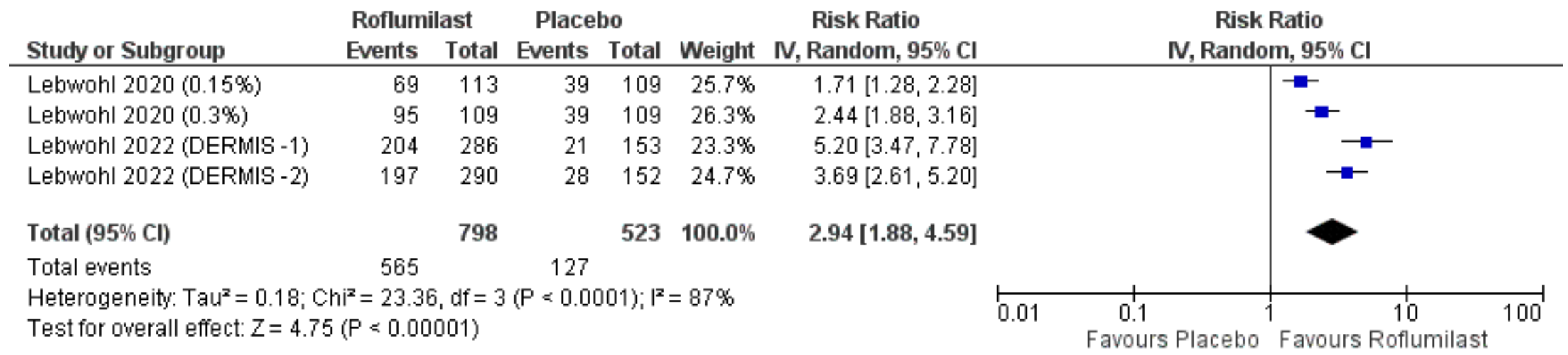


**Figure 13. Showing IGA score of 0 or 1 among patients with baseline intertriginous at week 8**

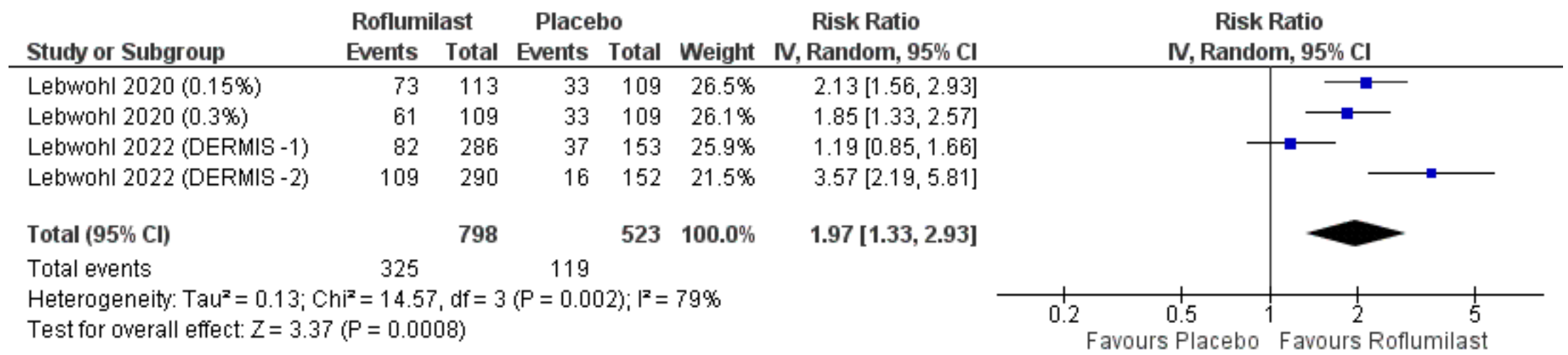


**Figure 14. Showing IGA score of 0 or 1 among patients with baseline intertriginous at week 4**

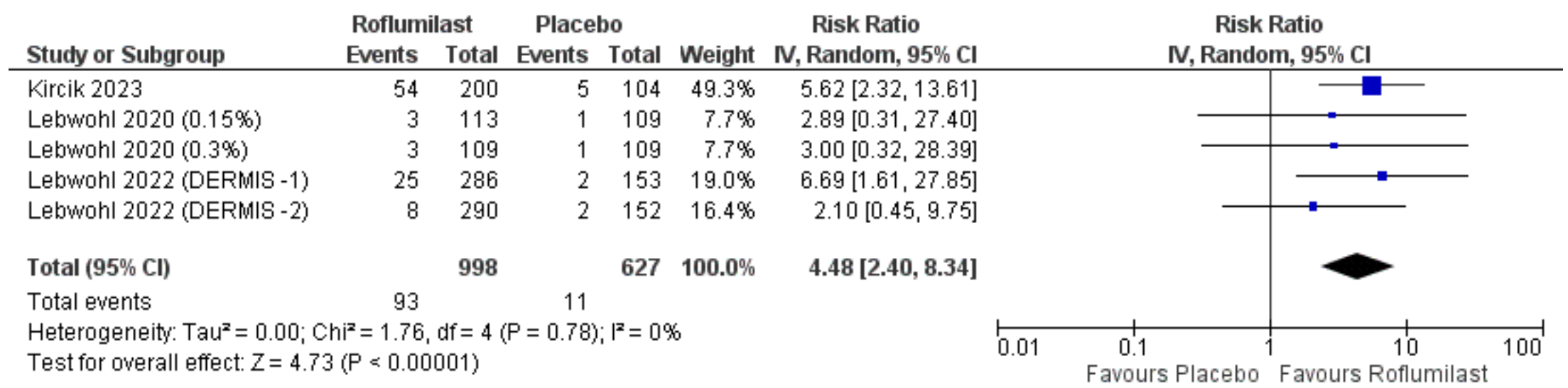


**Figure 15. Showing Percentage of patients achieving 75% reduction in severity & extent at week 2**

| Section and Topic | Item # | Checklist Item | Location Where Item is Reported |
|---|---|---|---|
| **TITLE** | | | |
| Title | 1 | Identify the report as a systematic review. | Title (Page 1). The title explicitly states "A Systematic Review and Meta-Analysis." |
| **ABSTRACT** | | | |
| Abstract | 2 | See the PRISMA 2020 for Abstracts checklist. | Abstract (Page 1). The abstract summarizes background, methods, results, and conclusions, aligning with PRISMA 2020 Abstracts checklist requirements. |
| **INTRODUCTION** | | | |
| Rationale | 3 | Describe the rationale for the review in the context of existing knowledge. | Introduction (Pages 2-4). The section discusses psoriasis prevalence, pathogenesis, current treatments, limitations of corticosteroids, and the potential of Roflumilast, providing context for the review. |
| Objectives | 4 | Provide an explicit statement of the objective(s) or question(s) the review addresses. | Introduction, last paragraph (Page 4). States: "We conducted this systematic review and meta-analysis to evaluate the current evidence on the effectiveness and safety of roflumilast for psoriasis." |
| **METHODS** | | | |
| Eligibility criteria | 5 | Specify the inclusion and exclusion criteria for the review and how studies were | Methods, Subsection 2.2 Inclusion and Exclusion Criteria (Page 5). Lists inclusion (RCTs comparing Roflumilast vs. placebo, patients ≥2 years, severity indices) and exclusion criteria (non-RCTs, non-English studies, etc.). Synthesis grouping is implied |

| | | | |
|---|---|---|---|
| | | grouped for the syntheses. | in Subsection 2.7 Statistical Analysis. |
| Information sources | 6 | Specify all databases, registers, websites, organisations, reference lists and other sources searched or consulted to identify studies. Specify the date when each source was last searched or consulted. | Methods, Subsection 2.1 Database Searching (Page 5). Lists Cochrane Central, PubMed, Scopus, Web of Science, searched up to March 2024. Reference lists are mentioned in Subsection 2.3 Study Selection (Page 5). |
| Search strategy | 7 | Present the full search strategies for all databases, registers and websites, including any filters and limits used. | Methods, Subsection 2.1 Database Searching (Page 5). Provides search terms with Boolean operators, e.g., "(Roflumilast OR 'daliresp') AND (Psoriasis OR 'psoriasis vulgaris' ...)." Notes no time constraints. Full search strings for each database are not provided |
| Selection process | 8 | Specify the methods used to decide whether a study met the inclusion criteria of the review, including how many reviewers screened each record and each report retrieved, whether they worked independently, and if applicable, details of automation tools used in the process. | Methods, Subsection 2.3 Study Selection and Data Extraction (Page 5). Describes use of EndNote and Rayyan, two independent reviewers for title/abstract and full-text screening, resolution by discussion or third reviewer, no automation tools mentioned. |
| Data collection process | 9 | Specify the methods used to collect data from reports, including how many reviewers collected data from each report, whether they worked independently, any processes for obtaining or confirming data from study investigators, and if applicable, details of automation tools used in the process. | Methods, Subsection 2.3 Study Selection and Data Extraction (Page 5). States data were manually extracted into a Google sheet by two independent reviewers, no mention of contacting investigators or automation tools. |
| Data items | 10a | List and define all outcomes for which data were sought. Specify whether all results that were compatible with each outcome domain in each study were sought, and if not, the methods used to decide which results to collect. | Methods, Subsection 2.4 Efficacy Outcomes and 2.5 Safety Outcomes (Page 6). Lists primary (IGA, PASI) and secondary outcomes (NRS, PSD, I-IGA), all relevant results sought (implied by meta-analysis inclusion criteria). |
| | 10b | List and define all other variables for which data were sought (e.g. participant and intervention characteristics, funding sources). Describe any assumptions made about any missing or unclear information. | Results, Subsection 3.2 Characteristics of the Studies (Page 7). Lists variables like year, population, baseline characteristics, study locations. Methods, Subsection 2.3 (Page 5), states assumptions for missing data (uniformity, exclusion of incomplete data). |
| Study risk of bias assessment | 11 | Specify the methods used to assess risk of bias in the included studies, including details of the tool(s) used, how many reviewers assessed each study and whether they worked independently, and if applicable, details of automa- | Methods, Subsection 2.6 Risk of Bias Assessment (Page 6). Describes use of Cochrane RoB1 tool, two independent reviewers, no automation tools. |

| | | | |
|---|---|---|---|
| | | tion tools used in the process. | |
| Effect measures | 12 | Specify for each outcome the effect measure(s) (e.g. risk ratio, mean difference) used in the synthesis or presentation of results. | Methods, Subsection 2.7 Statistical Analysis (Page 6). Specifies mean difference (MD) or standardized MD (SMD) for continuous outcomes, risk ratio (RR) for dichotomous outcomes, with 95% CI. |
| Synthesis methods | 13a | Describe the processes used to decide which studies were eligible for each synthesis (e.g. tabulating the study intervention characteristics and comparing against the planned groups for each synthesis (item #5)). | Methods, Subsection 2.7 Statistical Analysis (Page 6). States meta-analysis included outcomes from at least three studies, implying eligibility based on outcome availability. |
| | 13b | Describe any methods required to prepare the data for presentation or synthesis, such as handling of missing summary statistics, or data conversions. | Methods, Subsection 2.7 Statistical Analysis (Page 6). States missing statistics addressed by contacting authors or using available data, conversions per Cochrane guidelines. |
| | 13c | Describe any methods used to tabulate or visually display results of individual studies and syntheses. | Results, Subsections 3.1-3.6 (Pages 7-9). Mentions tables (Tables 1-3) and figures (Figures 1, 4-12) for study characteristics, forest plots, and safety outcomes. |
| | 13d | Describe any methods used to synthesize results and provide a rationale for the choice(s). If meta-analysis was performed, describe the model(s), method(s) to identify the presence and extent of statistical heterogeneity, and software package(s) used. | Methods, Subsection 2.7 Statistical Analysis (Page 6). Describes random-effects model, chi-square test, $I^2$ statistic, RevMan 5.4, rationale for conservative effect estimate. |
| | 13e | Describe any methods used to explore possible causes of heterogeneity among study results (e.g. subgroup analysis, meta-regression). | Methods, Subsection 2.7 Statistical Analysis (Page 6). Mentions sensitivity analysis for heterogeneity. Results, Subsections 3.4-3.5 (Pages 8-9), describe subgroup analyses. |
| | 13f | Describe any sensitivity analyses conducted to assess robustness of the synthesized results. | Methods, Subsection 2.7 Statistical Analysis (Page 6). Mentions sensitivity analysis. Results, Subsection 3.5 (Page 9), confirms sensitivity analysis for specific outcomes. |
| Reporting bias assessment | 14 | Describe any methods used to assess risk of bias due to missing results in a synthesis (arising from reporting biases). | Methods, Subsection 2.7 Statistical Analysis (Page 6). Describes funnel plots and Egger's test for publication bias. Results, Subsection 3.7 Reporting Bias Assessment (Page 9), notes infeasibility due to few studies. |
| Certainty assessment | 15 | Describe any methods used to assess certainty (or confidence) in the body of evidence for an outcome. | Methods, Subsection 2.8 Certainty Assessment (Page 6). Describes GRADE approach. Results, Subsection 3.4 Primary Outcomes (Page 8), rates IGA and PASI evidence as moderate. |
| **RESULTS** | | | |
| Study selection | 16a | Describe the results of the search and selection process, from the number of records identified in the search to the | Results, Subsection 3.1 Study Selection (Page 7). Describes search yielding 343 records, 205 screened, 50 full-text, 5 included, with Figure 1 (flow diagram). |

| | | | |
|---|---|---|---|
| | | number of studies included in the review, ideally using a flow diagram. | |
| | 16b | Cite studies that might appear to meet the inclusion criteria, but which were excluded, and explain why they were excluded. | Results, Subsection 3.1 Study Selection (Page 7). Lists common exclusion reasons (non-RCTs, non-placebo comparators, non-English) and refers to Supplementary File |
| Study characteristics | 17 | Cite each included study and present its characteristics. | Results, Subsection 3.2 Characteristics of the Studies (Page 7). Cites Lebwohl (2020), Lebwohl (2022), Kircik (2023), Snape (2016), NCT03392168, with characteristics in Tables 1-2. |
| Risk of bias in studies | 18 | Present assessments of risk of bias for each included study. | Results, Subsection 3.3 Risk of Bias and Quality Assessment (Page 7). Describes RoB1 results: two low risk, two unclear, one high risk, with specific issues noted. |
| Results of individual studies | 19 | For all outcomes, present, for each study: (a) summary statistics for each group (where appropriate) and (b) an effect estimate and its precision (e.g. confidence/credible interval), ideally using structured tables or plots. | Results, Subsections 3.4-3.6 (Pages 8-9). Presents RR, MD, CI for outcomes (IGA, PASI, NRS, PSD) in forest plots (Figures 4-12) and tables (Table 3). |
| Results of syntheses | 20a | For each synthesis, briefly summarise the characteristics and risk of bias among contributing studies. | Results, Subsections 3.4-3.5 (Pages 8-9). Summarizes studies contributing to IGA, PASI, and secondary outcomes, with risk of bias from Subsection 3.3. |
| | 20b | Present results of all statistical syntheses conducted. If meta-analysis was done, present for each the summary estimate and its precision (e.g. confidence/credible interval) and measures of statistical heterogeneity. | Results, Subsections 3.4-3.6 (Pages 8-9). Presents RR, MD, CI, P-values, $I^2$ for IGA, PASI, NRS, PSD, and safety outcomes. |
| | 20c | Present results of all investigations of possible causes of heterogeneity among study results. | Results, Subsections 3.4-3.5 (Pages 8-9). Describes subgroup and sensitivity analyses resolving heterogeneity for PASI, PSD, and I-IGA outcomes. |
| | 20d | Present results of all sensitivity analyses conducted to assess the robustness of the synthesized results. | Results, Subsection 3.5 (Page 9). Notes sensitivity analyses for IGA and NRS at week 4, confirming robustness. |
| Reporting biases | 21 | Present assessments of risk of bias due to missing results (arising from reporting biases) for each synthesis assessed. | Not Applicable. No assessment of reporting biases |
| Certainty of evidence | 22 | Present assessments of certainty (or confidence) in the body of evidence for each outcome assessed. | Not Applicable. |
| **DISCUSSION** | | | |
| Discussion | 23a | Provide a general interpretation of the results in the context of other evidence. | Discussion, Main Findings (Page 10). Interprets Roflumilast's effectiveness and safety compared to placebo, contextualized with psoriasis treatment challenges. |
| | 23b | Discuss any limitations of the | Discussion, Limitations (Page 10). Notes limited number of trials, unresolved hetero- |

| | | | |
|---|---|---|---|
| | | evidence included in the review. | geneity, and need for more RCTs. |
| | 23c | Discuss any limitations of the review processes used. | Discussion, Limitations (Page 10). Notes potential limitations from database coverage and manual data extraction errors. |
| | 23d | Discuss implications of the results for practice, policy, and future research. | Discussion, Conclusion (Page 10). Suggests Roflumilast's potential and need for further trials, implying research implications. |
| **OTHER INFORMATION** | | | |
| Registration and protocol | 24a | Provide registration information for the review, including register name and registration number, or state that the review was not registered. | Methods (Page 5). States registration with PROSPERO (CRD42024529658). |
| | 24b | Indicate where the review protocol can be accessed, or state that a protocol was not prepared. | Methods (Page 5). States protocol available via PROSPERO (https://www.crd.york.ac.uk/prospero/display_record.php?RecordID=429658). |
| | 24c | Describe and explain any amendments to information provided at registration or in the protocol. | Not Applicable. No amendments mentioned |
| Support | 25 | Describe sources of financial or non-financial support for the review, and the role of the funders or sponsors in the review. | Declarations, Funding and Acknowledgment (Page 11). Notes support from King Faisal University (Grant No. KFU250580), no role specified. |
| Competing interests | 26 | Declare any competing interests of review authors. | Declarations, Conflict of Interest (Page 11). States no conflicts of interest. |
| Availability of data, code and other materials | 27 | Report which of the following are publicly available and where they can be found: template data collection forms; data extracted from included studies; data used for all analyses; analytic code; any other materials used in the review. | Declarations, Data Availability (Page 11). States data, forms, extracted data, and code available on request; supplementary files include search strategy and excluded studies. |
